# DJ-1 promotes development of DEN-induced hepatocellular carcinoma and proliferation of liver cancer cells

**DOI:** 10.18632/oncotarget.14293

**Published:** 2016-12-27

**Authors:** Bijun Qiu, Junqi Wang, Yingxue Yu, Chao Zhen, Jinyang Gu, Wenjun Liu, Yankai Wen, Lili Chen, Yueqiu Gao, Qiang Xia, Xiaoni Kong

**Affiliations:** ^1^ Department of Liver Surgery, Renji Hospital, School of Medicine, Shanghai Jiao Tong University, Shanghai, China; ^2^ Department of Liver Diseases, Shuguang Hospital Affiliated to Shanghai University of Chinese Traditional Medicine, Shanghai, China; ^3^ School of Biomedical Engineering and Med-X Research Institute, Shanghai Jiao Tong University, Shanghai, China

**Keywords:** DJ-1, IL-6/STAT3, hepatocellular carcinoma, MHCC-97L

## Abstract

Chronic liver inflammation and injuries play a critical role in development of hepatocellular carcinoma (HCC). Parkinson disease (autosomal recessive, early onset) 7, encoding PARK7 protein (also called DJ-1), plays important roles in many carcinogenesis processes and is essential in modulating inflammation. However, whether DJ-1 is involved in HCC development remains largely unknown. To determine the effect of DJ-1 on HCC development, we accessed the correlation of hepatic DJ-1 expression with overall survival (OS) and TNM stage in 96 HCC patients and found a significant inverse correlation between DJ-1 expression and OS. By adopting a classic diethylnitrosamine (DEN)-induced murine HCC model, DJ-1 knockout (KO) mice displayed reduced tumorigenesis and cell proliferation, accompanied by decreased hepatic inflammation and IL-6/STAT3 activation. Furthermore, after an acute DEN challenge, DJ-1 KO mice showed significant decreases in liver injury, hepatocyte proliferation and DNA damage. In a human HCC cell line (MHCC-97L), cancer cell proliferation was induced by overexpression of DJ-1 and is related to oncogenic signaling of MAPKs and AKT. Induction of DJ-1 may serve as a novel regulator for HCC cell proliferation and HCC development possibly through enhanced MAPK signaling and inflammation.

## INTRODUCTION

Hepatocellular carcinoma (HCC) is the third most common cause of cancer-related deaths worldwide and the major global health problem currently [1, 2]. Due to lack of effective diagnostic and therapeutic approaches, the mortality rate of HCC in most developing countries almost equals the incidence rate [3, 4]. The studies about the underlying mechanisms of HCC are urgent. Inflammation appears to be one of causal factors in HCC development as over 90% of HCC cases arise in patients with chronic liver injury and inflammation.

DJ-1 was initially found to be an oncogene promoting Ras-inudced cell transformation [5], and later on was demonstrated to associate with early-onset Parkinson's disease [6, 7]. Accumulating evidence has shown amplification of DJ-1 in many types of cancers [8–10]. As an oncogene, DJ-1 drives AKT-mediated cell survival, activates mTOR and MAPK and inhibits PTEN to protect cell against hypoxia-induced cell death [11–13]. In addition, there are abundant studies supporting the notion that amplification of DJ-1 plays a cytoprotective role by eliminating oxidative stress via oxidizing itself and/or stabilizing Nrf2, a master regulator of antioxidant transcriptional responses [14, 15]. Our recent study indicates that DJ-1 is pivotal in directing NADPH-ROS production in macrophages, and subsequently affects the inflammatory response in sepsis [16]. However, whether DJ-1 contributes to HCC development is largely unknown.

Signal transducer and activator of transcription 3 (STAT3) is constitutively activated in many types of cancers serves as a poor prognostic indicator and oncopromoter [17]. Activated STAT3 protects cell death induced by growth factor deprivation in human squamous carcinoma cells [18]. STAT3 is key mediator downstream of EGFR active mutations in non-small-cell lung cancer [19]. Notably, as the major inflammation mediator, IL-6 is a dominant activator of STAT3 to promote cancer progression [20–22].

Given the tight association between HCC and inflammation, the capacity of DJ-1 to modulate inflammation and the oncogenic role of DJ-1 in many cancer types, we hypothesized that DJ-1 contributes to HCC development by stimulating inflammation response. In present study, we demonstrate a significant inverse correlation between DJ-1 and overall survival (OS) in HCC patients, and an attenuated HCC development in DJ-1 KO mice compared to WT mice in a DEN-induced HCC murine model. This may be attributed to reduced immune cell infiltration, IL-6 induction and STAT3 activation in DJ-1 KO mice. In addition, we also show that DJ-1 has a direct positive effect on cell proliferation in human hepatocellular cell lines. These findings suggest that DJ-1 promotes HCC development and might be a novel therapy target for HCC.

## RESULTS

### DJ-1 expression is negatively correlated to HCC prognosis

To determine whether DJ-1 is associated to HCC development, we first examined the DJ-1 levels in tumors of 96 HCC patients by immunohistochemistry (IHC). Representative images of high or low expression of DJ-1 in HCC tumor tissues were shown in Figure 1A. DJ-1 protein was mainly expressed in the cytoplasm of tumor cells. We then compared the DJ-1 levels in tumor and matched normal tissues by western and found that DJ-1 expression was higher in tumor tissue (Figure 1B). We further evaluated the correlation between DJ-1 expression and clinicopathologic variables and found that DJ-1 expression was negatively correlated with overall survival (OS) in HCC patients (Figure 1C). However, the association between DJ-1 levels and HCC TNM stage was not significantly although HCCs with low DJ-1 levels displayed more stage I and fewer stage III cases compared with the DJ-1 high counterparts (Figure 1D). Given that AFP and tumor size are two independent prognostic factors for OS in HCC, we then categorized these 96 HCC patients into four subgroups, AFP>200ng/ml, AFP<200ng/ml, tumor size>5cm and tumor size<5cm. As shown in Figure 1E, high DJ-1 expression was significantly associated with poor OS in subgroups of AFP<200ng/ml or tumor size>5cm. However, DJ-1 expression was not significantly although negatively correlated with OS in subgroups of AFP>200ng/ml or tumor size<5cm (Figure 1E). Probably due to limited clinical sample size in these subgroups, The correlations of DJ-1 with other variables are listed in Supplementary Table 1. These findings suggest that DJ-1 is possibly involved in HCC progression and can serve as a prognostic marker of HCC.

**Figrue 1:**
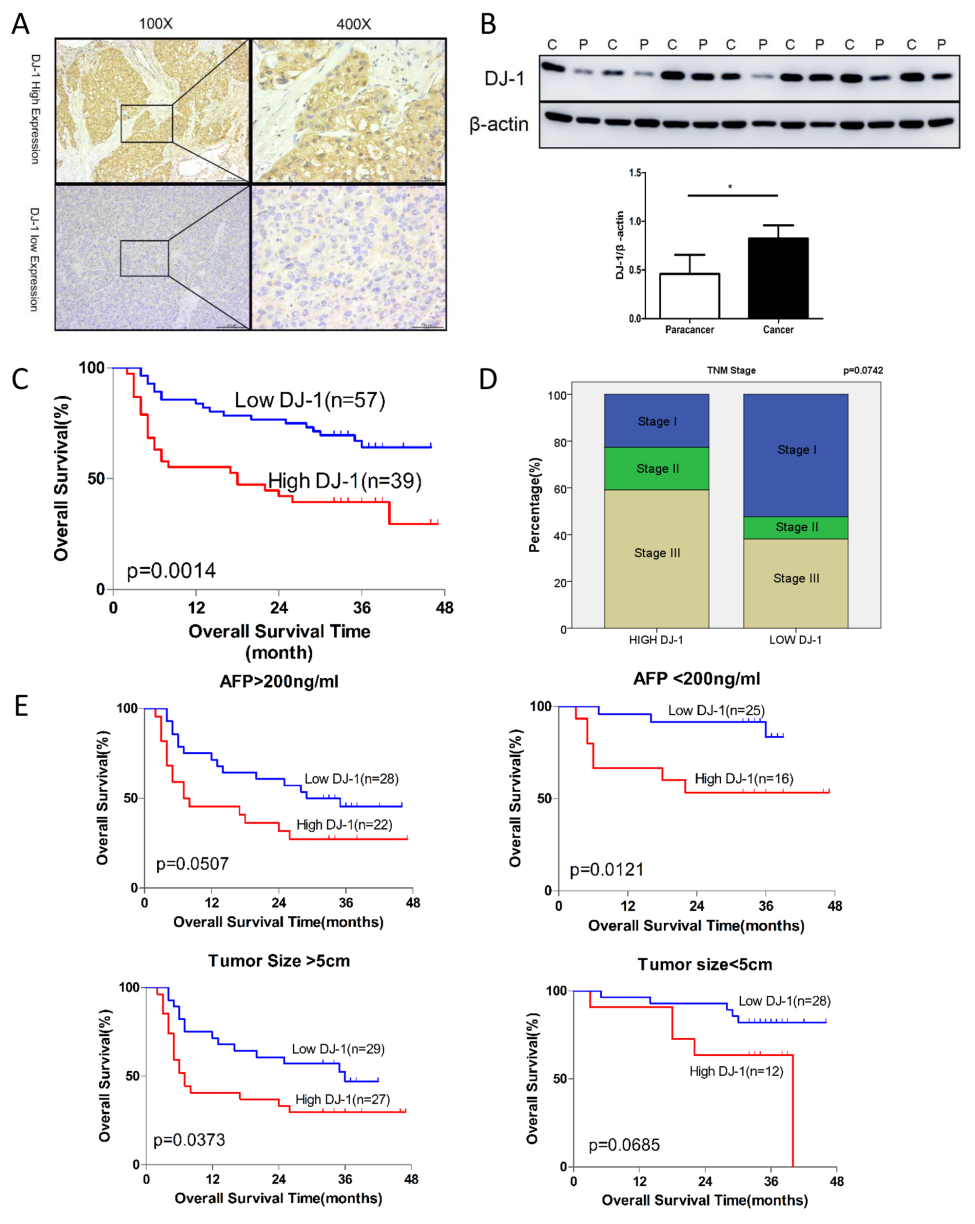
Induction of DJ-1 expression is inversely correlated with survival rate in patients with HCC **A**. Expression of DJ-1 in hepatpcellular carcinoma tissue was examined with immunohistochemistry. **B**. Expression of DJ-1 in tumor tissue and paratumor tissue was examined with immunoblotting. Bar graph shows quantification results, data are represented as mean +/− SEM **C**. The prognostic significance of DJ-1 for HCC patients assessed by log-rank test. **D**. Correlation of DJ-1 expression with TNM stages in HCC patients. **E**. The prognostic significance of DJ-1 for subgroup of HCC patients assessed by log-rank test.

### DJ-1 depletion attenuates hepatoellular carcinoma development in a DEN-induced HCC mouse model

**Figure 2:**
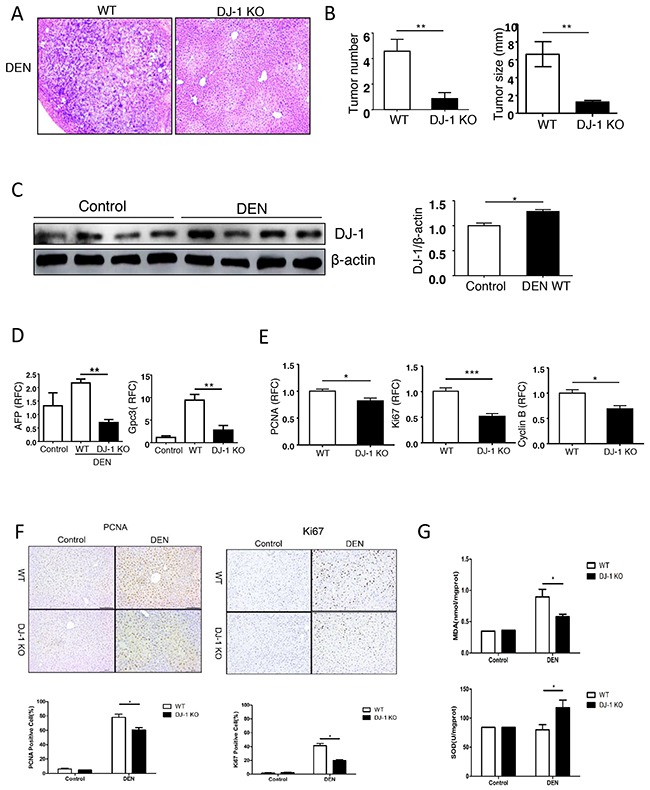
Loss of hepatic DJ-1 reduces hepatocyte proliferation, tumor development and oxidative stress in a DEN-induced mouse model of HCC **A**. Representative microscopic images of HCC stained with H&E in DJ-1 KO and WT mice. **B**. Tumor numbers and tumor sizes were assessed 10-months after DEN injection in WT mice and DJ-1 KO mice. Data are represented as mean +/− SEM **C**. Hepatic DJ-1 expression 10-month after DEN treatme compared to control group analyzed by Immunoblotting. Bar graph shows quantification results, data are represented as mean +/− SEM **D**. Clinical indicators AFP, Gpc3 for HCC were analyzed by qRT-PCR. Data are represented as mean +/− SEM **E**. Hepatocytes proliferation with PCNA, Ki67, CyclinB gene expression were analyzed by qRT-PCR. Data are represented as mean +/− SEM **F**. Hepatocytes proliferation with PCNA and Ki67 protein expression were analyzed by IHC. Bar graph shows quantification results, data are represented as mean +/− SEM G. MDA and SOD was measured in liver tissue. Data are represented as mean +/− SEM.

To further investigate the role of DJ-1 in HCC development, we adopted DJ-1 deficient mice (DJ-1 KO) and induced HCC using a traditional DEN mouse model. This model incorporates chronic liver injury with inflammation and mimics several features of the microenvironment in which the majority of human HCCs arise [23]. Male WT mice and DJ-1 KO mice received a single DEN (25mg/kg) injection intraperitoneally at 14 days postpartum [24, 25]. 10-month after this single injection, mice were sacrificed and the presence of HCC were examined by H&E staining (Figure 2A). Compared to WT mice, DJ-1 KO mice displayed a significant decrease in both tumor number and size (Figure 2B). Meanwhile, we found that the hepatic DJ-1 protein level was slightly but significantly increased in WT mice after 10-month DEN treatment compared to the Sham group (Figure 2C). Notably, In agreement with attenuated HCC development in DJ-1 KO mice, the expression of clinical HCC indicators AFP and Gpc3 was also reduced compared with WT mice (Figure 2D). Since excessive proliferation of hepatocytes is a hallmark of HCC development [26], we then detected the main proliferation markers, PCNA, Ki67 and Cyclin B, in the liver tissues 10-month post DEN challenge and found that all those proliferation markers significantly decreased in DJ-1 KO mice compared with WT mice (Figure 2E). Consistently, immunochemistry staining of PCNA and Ki67 showed more proliferative cells in WT mice than that in DJ-1 KO mice (Figure 2F). Given that DEN induced liver injury is associated with induction of ROS and DNA damage, we then measured MDA and SOD in liver tissue and revealed that the level of oxidative stress was higher in WT mice than that in DJ-1 KO mice (Figure 2G). However, TUNEL staining showed very few signals in both genotypes, probably due to the resolution during the chronic DEN model (Supplementary Figure 1). Together, these data revealed that DJ-1 deficiency protected mice from developing severe HCCs.

### Impaired IL-6/STAT3 activation may account for attenuated HCC development in DJ-1 KO mice

**Figure 3:**
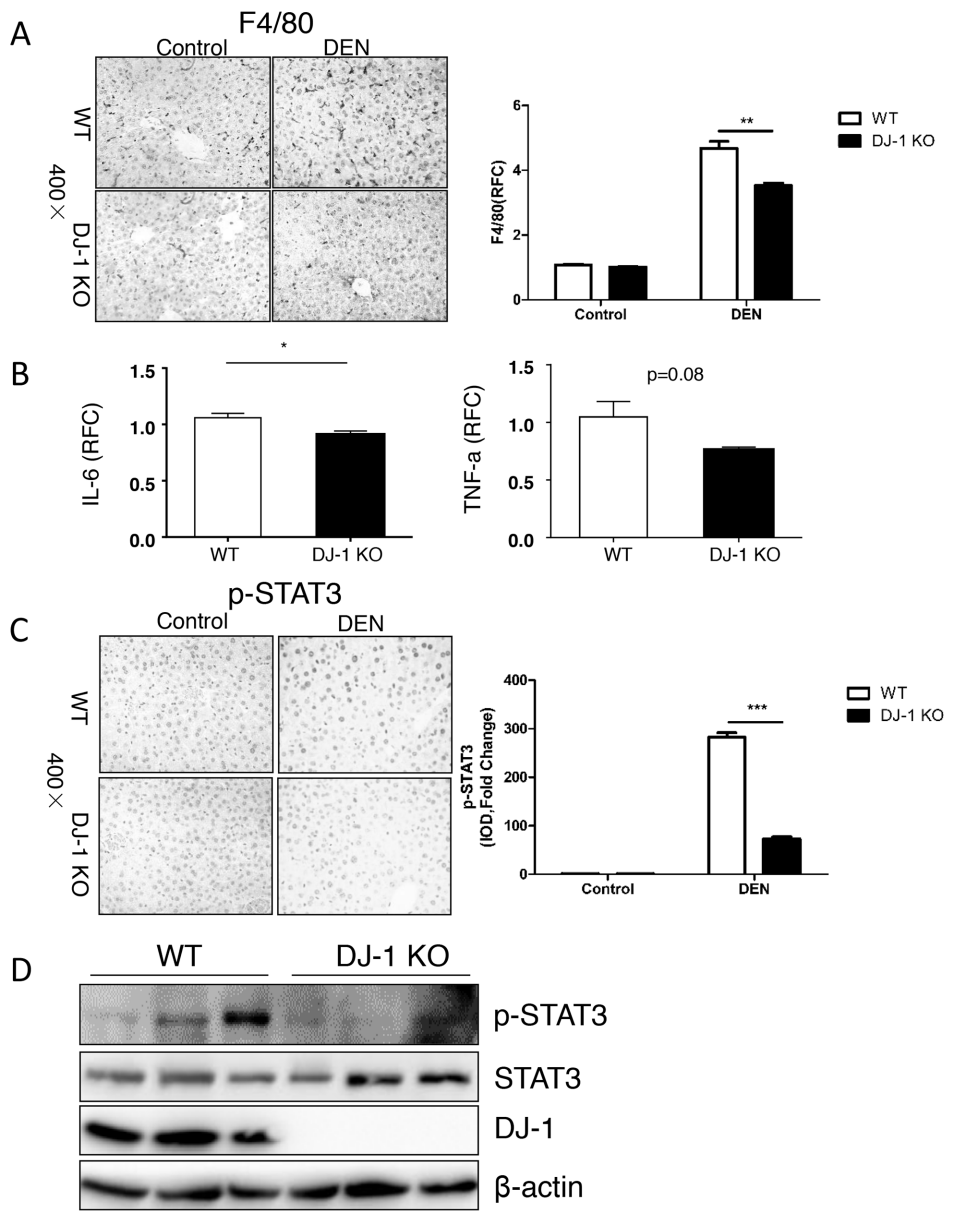
Hepatic DJ-I deficiency inhibits activation of IL-6/STAT3 signaling in mice with HCC **A**. F4/80 staining for macrophages was performed in DJ-1 KO and WT mice treated with DEN. Bar graph shows quantification results, data are represented as mean +/− SEM **B**. IL-6 and TNF-α mRNA level was measured in DEN treated DJ-1 KO and WT mice by qRT-PCR. Data are represented as mean +/− SEM **C**. p-STAT3 IHC staining was performed in DJ-1 KO and WT mice treated with DEN. Bar graph shows quantification results. data are represented as mean +/− SEM **D**. Western blot analysis was performed for the indicated protein from the DEN treated DJ-1 KO and WT mice.

Our previous study indicates that DJ-1 modulates inflammation in innate immune system [16]. To examine the inflammation status, we stained F4/80, a typical marker of macrophage, and found that fewer infiltrated macrophages in the liver of DJ-1 KO mice compared to WT mice (Figure 3A), suggesting lower hepatic inflammation in DJ-1 KO mice. Considering that macrophage is a major cell source of IL-6 and IL-6 plays an important role in HCC development [27–29], we then detected IL-6 level in the liver of WT and DJ-1 KO mice 10-month after DEN treatment. As shown in Figure 3B, significantly less IL-6 expression was detected in DJ-1 KO mice compared to WT mice. We also observed a TNF-a decrease although not significant in DJ-1 KO mice (Figure 3B). Given that IL-6 can activate downstream STAT3, a key oncogene constitutively activated in many cancer including HCCs and contributing to cancer cell proliferation and survival [30, 31] [32, 33] [34, 35], we examined hepatic STAT3 activation in both genotypes 10-month post DEN injection. In agreement with reduced IL-6 expression, DJ-1 KO mice showed impaired STAT3 activation as evidenced by attenuated phosphorylation of STAT3 through both immunochemistry staining and immunoblotting assays (Figure 3C and 3D). These findings suggest that DJ-1 may contribute to HCC development by modulating inflammation responses and in turn enhancing IL-6/STAT3 signaling.

### DJ-1 knockout mice are protected from acute DEN liver damage

**Figure 4:**
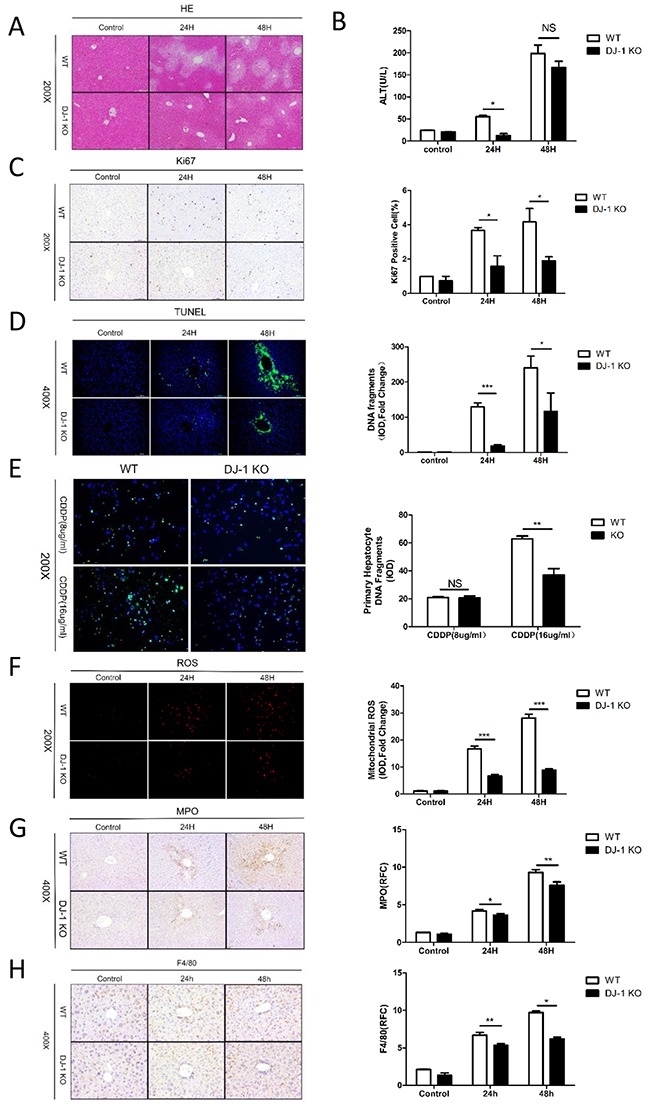
DJ-1 aggravates liver injunry, DNA damage and production of ROS in DEN acute model **A**. Representative microscopic images of tissue damage and inflammatory changes stained with H&E in DJ-1 KO and WT mice. **B**. Serum ALT levels of liver injury were detected in DJ-1 KO and WT mice. Data are represented as mean +/− SEM. **C**. Hepatocytes proliferation marker Ki67 expression was analyzed by IHC in DJ-1 KO and WT mice. Bar graph shows quantification results. data are represented as mean +/− SEM **D**. Hepatocyte cell death marker TUNEL expression was analyzed by immunofluorescence staining in DJ-1 KO and WT mice. Bar graph shows quantification results. Data are represented as mean +/− SEM **E**. TUNEL expression was analyzed by immunofluorescence staining in DJ-1 KO and WT primary hepatocyte. Bar graph shows quantification results. Data are represented as mean +/− SEM **F**. ROS was analyzed by immunofluorescence staining of Mitosox. Bar graph shows quantification results. Data are represented as mean +/− SEM **G**. Neutrophil was analyzed by IHC of MPO. Bar graph shows quantification results. Data are represented as mean +/− SEM **H**. Macrophage was analyzed by IHC of F4/80. Bar graph shows quantification results. data are represented as mean +/− SEM

The initial cellular damage is a known important reason of the incipient development of tumors in the DEN model. Therefore, we assessed liver damage, hepatic proliferation and hepatic cell death in both genotypes in an acute DEN injury model (a short term exposure of a high dose of DEN (200 mg/kg BW)). H&E staining revealed more severer tissue injuries mainly surrounding the portal tracks of the liver in WT mice compared to DJ-1 KO mice (Figure 4A). DJ-1 depletion exhibited greater hepatic protection as determined by significant lower ALT levels 24h post DEN treatment (Figure 4B). Ki67 staining displayed significantly reduced proliferation in DJ-1 KO mice when compared with WT mice (Figure 4C). Notably, we observed a greater hepatocyte death especially surrounding the portal tracks as assessed by terminal deoxynucleotidyl transferase dUTP nick-end labeling (TUNEL) immunofluorescence staining in WT mice animals compared with DJ-1 mice (Figure 4D), suggesting the protective role of DJ-1 deficiency in DEN-induced DNA damage. To further confirm this *in vivo* observation, we isolated primary hepatocytes and stimulated them with cisplatin at the concentration of 8ug/ml and 16ug/ml to induce DNA damage, followed with TUNEL assays. In line with the *in vivo* results, primary hepatocytes with DJ-1 depletion showed reduced DNA damage compared to WT hepatocytes (Figure 4E). These findings strongly suggest that, compared to WT mice, the diminished HCC development in DJ-1 KO mice may be attributed to decreased liver injury and hepatocyte proliferation after DEN treatment. Given that ROS mainly contribute to DEN-induced DNA damage, we further compared ROS induction in both genotypes. Corresponding to the less DNA damage in DJ-1 KO mice, attenuated hepatic ROS induction was detected in DJ-1 KO mice compared to WT mice as evidenced by mitoSOX Red staining (Figure 4F). Given that CYP2E1 mediates DEN metabolism and accounts for DEN-induced ROS production, we then examined hepatic CYP2E1 expression and found that there was no significant expression difference between in WT and KO mice (Supplementary Figure 2). Meanwhile, we also accessed hepatic inflammation by MPO and F4/80 staining. As shown in Figure 4G and 4H, significant less hepatic infiltration of neutrophils and macrophages was observed in DJ-1 KO mice.

### DJ-1 affects cell proliferation and oncogenic pathway activation in MHCC-97L cell line

**Figure 5:**
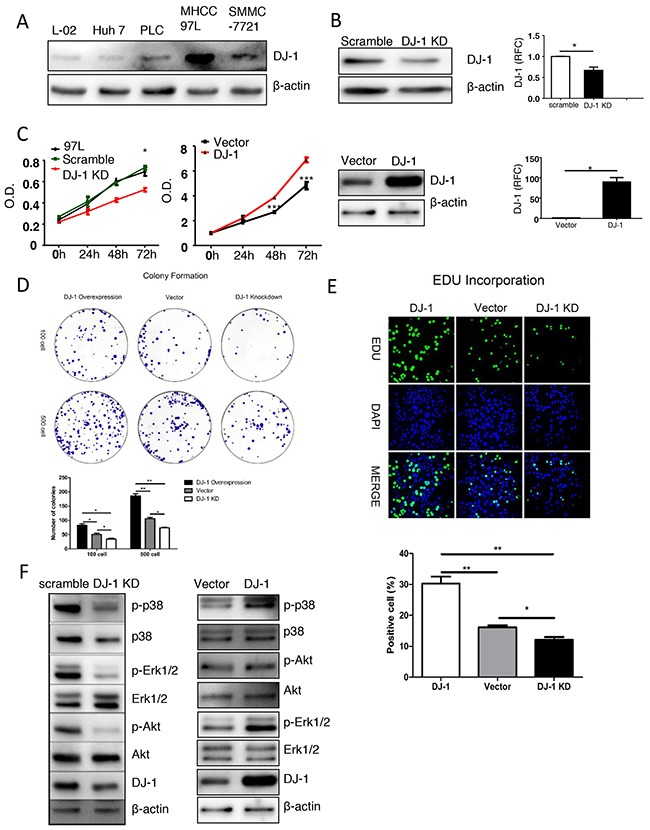
DJ-1 affects cell proliferation ad oncogenic pathway activation in MHCC-97L cell line **A**. Western blot analysis showing the protein expression of DJ-1 in different human cancer cell lines. **B**. mRNA and protein expression of DJ-1 in DJ-1 KD-97L cells, DJ-1 overexpression 97L cells and Control cells were detected. Data are represented as mean +/− SEM. **C**. The proliferation rates of MHCC-97L cells, Control cells and DJ-1 KD-97L cells were measured by MTT assay. **D**. Colony formation of DJ-1 KD-97L cells,DJ-1 overexpression 97L cells and Control cells. Bar graph shows quantification results. Data are represented as mean +/− SEM. **E**. EDU incorproation measured in DJ-1 KD-97L cells, DJ-1 overexpression 97L cells and Control cells and bar graph shows quantification results. Data are represented as mean +/− SEM.**F**. Proliferation related signaling pathways were detected by Western blot analysis using the cell lysates of DJ-1 KD-97L cells, DJ-1 overexpression 97L cells and Control cells.

Next, we started to explore the oncogenic function of DJ-1 in human HCC cell lines *in vitro*. We first measured expression of DJ-1 in different human liver cancer cell lines compared with normal human liver cell line L-02 by western blotting (Figure 5A). Among them, MHCC-97L was selected for further experimental studies due to high DJ-1 expression. We transfected MHCC-97L cells with plasmids carrying a DJ-1-shRNA or scramble sequence to knock down DJ-1 expression. Stable transfectants were selected and knockdown efficiency was confirmed by immunoblotting and qRCR (Figure 5B). MTT assays showed significant reduced proliferation in cells with DJ-1 knockdown compared to cells expressing scramble sequence or mock (Figure 5C). In line with the loss-of-function of DJ-1, overexpressing DJ-1 in MHCC-97L cells resulted in significant increased cell proliferation as indicated by MTT assays (Figure 5C). To further confirm the role of DJ-1 in promoting cancer cell proliferation, we performed colony formation assays and Edu incorporation. Consistent with the MTT results, DJ-1 knockdwon caused reduced proliferation as indicated by less colony formation and Edu incorporation (Figure 5D and 5E). In contrast, DJ-1 overexpression enhanced proliferation (Figure 5D and 5E). Given that activation of several oncogenic pathways such as MAPKs (p38, Erk) and AKT contributes to cancer cell proliferation, we then examined the activation levels of those oncogenic pathways. As shown in Figure 5F, in response to DJ-1 knockdown or overexpression, the phosphorylation signals of p38, Erk and Akt were either greatly reduced or increased respectively in MHCC-97L cells. Therefore, our findings suggest the tumor promoting potential of DJ-1 in HCC by activating multiple oncogenic pathways.

## DISCUSSION

The relationship between inflammation and cancer was first hypothesized by a German pathologist, Rudolf Virchow, in the early 1860s [36]. So far, it has been widely accepted that chronic inflammation is one of important contribution components in the pathogenesis of many types of malignancies [37–39]. During inflammation, immune cells infiltrate into injured sites and produce a host of cytokines and chemokines that further propagate a localized inflammatory response and enhance the growth and survival of premalignant cells. IL-6 is a well-known important proinflammatory cytokine tightly linked to increased cancer cell proliferation and tumor progression [40, 41]. Through binding with IL-6R, IL-6 activates JAK-STAT3 signaling pathway and in turn confers tumor cells proliferation advantages. In present study, we demonstrate that DJ-1 KO mice had decreased inflammation (hepatic infiltration of macrophages), IL-6 and pSTAT3 levels in response to DEN administration, which may be attributed to attenuated HCC development in DJ-1 KO mice.

The oncogenic role of DJ-1 has been investigated in many human cancers, including human HCC. A previous study has demonstrated that DJ-1 is highly expressed in HCC tissues compared to matched normal tissues and may serve as a candidate prognostic biomarker of HCC [42]. Another study has uncovered the association of DJ-1 expression with tumor invasion and metastasis [43]. Consistent with these clinical studies, we showed inverse correlation of DJ-1 with OS in HCC patients. However, those two previous studies mainly focused on the clinical analysis of HCC samples, but the mechanism by which DJ-1 contributes to HCC initiation and development remains largely unknown. By exploiting a DEN-induced HCC mouse model, we demonstrate that HCC initiation and development was severely impaired in DJ-1 KO mice compared to WT mice. Mechanistically, DJ-1 depletion results in decreased ROS induction, liver injury, injury mediated hepatic inflammation and subsequent STAT3 activation after DEN treatment. *In vitro* functional experiments also showed that DJ-1 itself promotes HCC proliferation by activating multiple oncogenic pathways, such as MAPKs and AKT. Given the well-known inhibition of DJ-1 to PTEN and the inhibitory role of PTEN on MAPKs and AKT [44, 45], it is conceivable that DJ-1 mediated activation of MAPKs and AKT pathways may through PTEN inhibition.

In regard to the highly correlation with tumors and multiple biological regulation functions, DJ-1 has been suggested to be a diagnostic marker and even prognostic factor for cancers [46, 47]. In hepatocarcinoma, we demonstrated abundant DJ-1 correlates to severe HCC development with strong proinflammatory cytokine IL-6 production which implies a clinical value for early diagnosis of hepatocarcinoma in the stage of liver inflammation.

## MATERIALS AND METHODS

### Patient samples

96 patients, who were diagnosed as hepatocellular carcinoma (HCC), were enrolled in this study. Patient samples were obtained following informed consent according to established protocols approved by the Ethics Committees of the Renji Hospital (Shanghai,China). DJ-1 immunohistoch- emistry was performed on one millimeters sections obtained from a tissue micro-array (TMA) comprising hepatocellular carcinoma tumoral material. The protocol is described below. Tumor specimens embedded in the TMA encompassed tissue material derived from the 96 patients with known clinical data after hepatectomy (Supplementary Table 1).

### Animals

DJ-1 knockout mice (stock#006577) were purchased from the Jackson Laboratory (Bar Harbor, Maine, USA). C57BL/6 mice were purchased from SLAC laboratory (Shanghai, China). All mice were bred and maintained at an animal facility under specific pathogen free condition. All studies with animal were under the criteria outlined in Guide for the Care and Use of Laboratory Animals, which was approved by Bioethics Committee School of Medicine, Shanghai Jiao Tong University.

### Diethylnitrosamine (DEN)-induced hepatocellular carcinoma model

14-day old WT and DJ-1 KO male mice were intraperitoneally injected with DEN (25mg/kg BW) (sigma, # MKBL 4199V). Mice were subsequently sacrificed 10 months after DEN injection. The acute DEN model was studied with 8-week old WT mice and DJ-1 KO mice intraperitoneally injected with 200mg/kg BW DEN.

### Biochemical analysis

Peripheral blood sera were harvested from mice injected with DEN, then the blood was centrifuged at 5000rpm for 10 min at 4°C, the supernatant was transferred into a new tube. Serum Alanine transaminase (ALT) levels were assessed according to the manufacturer's instructions from Nanjing Jiancheng Bioengineering Institute (China).

### Western blotting

Liver samples were homogenized in RIPA buffer (Thermo Scientific, Rockford, IL) containing a protease inhibitor cocktail (Calbiochem, Raleigh, NC). Protein quantification was performed with a BCA protein assay kit (Thermo Scientific, Rockford, IL). Protein samples were separated by 10% SDS-PAGE. The gel was transferred to a nitrocellulose membrane and probed with antibodies against indicated antigens, followed by corresponding secondary antibodies coupled with horseradish peroxidase. Detection and quantification of protein bands were performed using the ChemiDocTM XRS+ System with Image LabTM Software (Bio-Rad). Primary antibodies were DJ-1 (abcam), b-actin (Sigma), p-p38, p38, p-pAkt, Akt, p-Erk1/2, Erk1/2, STAT3, p-STAT3 (Cell Signaling Technology).

### Histopathology and immunohistochemistry

For liver histological assay, mice liver tissues were embedded with paraffin, 5um-thick sections were performed with immunohistochemistry staining. Tissue microarray and sections from WT mice and DJ-1 KO mice were dewaxed, rehydrated and subjected to epitope retrieval step in citric acid solution or Tris-EDTA buffer. Endogenous peroxidase activity was blocked with 3% hydrogen peroxide. Tissue microarray and sections were blocked with 10% bovine serum albumin and incubated at 4°C overnight with primary antibody of DJ-1(1:1000, Abcam, Cambridge, MA),F4/80 (1:100,AbD Serotec, Oxford, UK), p-STAT3 (1:50 Cell Signaling, Boston, MA), Ki67 (1:100, Abcam, Cambridge, MA), PCNA (1:100 Cell Signaling, Boston, MA) and MPO (BioCare Medical, Concord, CA), subsequently stained with DAB substrate and counterstained with hematoxylin. Every tissue sections on the Microarray and six to ten images from random fields in each section were taken with a light microscope (Axio Imager A1; Zeiss). For human DJ-1, immunohistochemical expression in HCC tissue was semi-quantitatively assessed using immunoreactive score (IRS). IRS can range from 0 to 12 and two classes were defined for the study: low expression staining (corresponding to IRS = 0-6) versus high expression staining (corresponding to IRS = 7-12). Briefly, assessment of IRS is based on the proportion of stained cells which is scored from 0 to 4, multiplied by the intensity of staining, ranging from 0 to 3: no positive cells (0), < 25% of positive cells (1), 25-50% of positive cells (2), 51-75% of positive cells (3), > 75% of positive cells (4), no color reaction (0), mild reaction (1), moderate reaction (2), intense reaction (3) is used to calculate this score.

### qRT-PCR assays

Total RNA was extracted by using RNeasy Mini Kit (Qiagen, Valencia, CA), according to the manufacturer's instructions. Total RNA (1ug) was used for cDNA synthesis (Thermo Scientific, Rockford, IL). Gene expression levels were detected by SYBR-green based Quantitative RT-PCR (TaKaRa Biotechnology, Dalian, China) with gene specific primers listed below: mouse PCNA primers, forward: GTGGAGCAACTTGGAATCCC; reverse: GGTTACCGCCTCCTCTTCTT, Mouse Ki67 primers, forward: TAACTTGCGCTGACTGGACC; reverse: GGAGAAGCCTCTCGGTGAAG, mouse Cyclin B primers, forward: AAGACTCCCTGCTTCCTGTT; reverse: CGGCCTTAGACAAATTCTGA, mouse IL-6 primers, forward: TGTTCTCTGGGAAATCGTGGA; reverse: TTTCTGCAAGTGCATCATCGT, mouse AFP primers, forward: TTTCCAGAACCTGCCGAGAG; reverse: CAGAATGGCTGGGGCATACA, Mouse Gpc3 primers, forward: TGATACCCTGTGCTGGAACG; reverse: GGCTCAGGGCCCTTCATTTT, human DJ-1 primers, forward: TCTGAGTCTGCTGCTGTGAA; reverse: AGTAGGACCTGCACAGATGG.

### Staining of ROS

For ROS staining, mice liver tissues were embedded with OCT, 8um-thick sections were cut by freezing microtome. The sections should be balanced 10 minutes in 4°C and rewarming 15 minutes in room temperature. Then rinse the sections three times in icy PBS to clean up the OCT. Add mitosox 2uM and incubating in 37°C for 30 minutes. Finally rinse the sections three times in PBS. Add DAPI and mounting then observe under fluorescence microscope.

### Tissue staining of TUNEL

Dewax and rehydrate tissue section according to standard protocols by heating at 70°C followed by washing in xylene and rehydration through a graded series of ehanol and double dist. Water. Then incubate tissue section for 15minutes at 37°C with proteinase K working solution (proteinase K:buffer solution=1:1000). Rinse sections three times with PBS. ADD 50ul TUNEL reaction mixture on sample. Incubate sections in a humidified atmosphere fo 60 minutes at 37°C in the dark. Rinse section three times with PBS. Samples can directly be analysed under a fluorescence microscope.

### Primary hepatocyte staining of TUNEL

Fix air dried cell sample with a freshly prepared 4% paraformaldehyde for 1 hour at room temperature. Rinse slides with PBS. Incubate in 0.1%TritonX-100 in 0.1% sodium citrate for 2 minutes on ice. ADD 50ul TUNEL reaction mixture on sample. Incubate sections in a humidified atmosphere fo 60 minutes at 37°C in the dark. Rinse section three times with PBS. Samples can directly be analysed under a fluorescence microscope.

### Primary hepatocyte isolation

Primary hepatocyte were obtained by using perfusion method. In brief, liver was perfused via portal vein with EGTA buffer (5.4mM KCl, 0.44mM KH2PO4, 0.338mM Na2HPO4, 137mM NaCl, 25mM Tricine and 0.5mM EGTA) 40ml for 5 min, followed by another perfusion with Gey's balanced salt solution (GBSS)(2mM CaCl2, 1mM MgCl2, 0.285mM MgSO4, 5mM KCl, 0.20mM KH2PO4, 27mM NaHCO3, 120mM NaCl, 0.8mM Na2HPO4, 5.6mM D-Glucose) containing 0.075% collagenase IV (Sigma) 40ml for 20min. After that, liver was dissociated with digestion GBSS buffer with collagenase IV (0.008%) for 30 min at 37°C and filtered with a 70-μm Nylon cell strainer. After centrifuged at 500 rpm for 5 min, the hepatocyte pellets were seeded at 24-well plates for further experiments.

### DJ-1 knockdown and overexpression in human cancer cell line

To knock down DJ-1 in MHCC-97L cells, a DJ-1 shRNA oligo and a scramble shRNA oligo were synthesized and cloned into the pSilencer-3.1-Hygro (Ambion). The sequence of the inserted DJ-1 shRNA oligo is: GATCCGCTAAAGGAGCAGAGGAAATTTCAAGAGAATTTCCTCTGCTCC

TTTAGTTTTTTGGAAA, and the scramble oligo sequence is: GATCCGATCTCTTCTGGTATTA

GACTCAAGAGATCTAATACCAGAAGAGATCTTTTTTGGAAA. After transfection, cells were selected with hygromycin (1000ug/ml). Stable clones were selected and DJ-1 knockdown efficiency was detected by qRT-PCR and western blotting. DJ-1 cDNA were cloned into the mammalian expression vector pcDNA^TM^3.1/myc-His(-) A to generate pcDNA 3.1-DJ-1.

### MTT assay

For cell viability detection, MHCC-97L cells were planted into 96-well plates at 10^3^ cells per well. MTT assay was performed at 0h, 24h, 48h, 72h after cell adherence. Briefly, 20ul of 5mg/ml Thiazolyl Blue Tetrazolium Bromide (MTT, sigma,#M2128) was added into medium and the plate was placed in 37°C incubator for 4h. Then, the medium was gently discarded and 150ul sterile DMSO was added to dissolve the formazan crystal. All process was performed in the dark. Plates were immediately read on a microplate reader at the wavelength of 490nm and 630nm.

### EDU incorproation

Seed cells at 96-well plate each well about 3*10^4^ cells. Incubate cells with EDU working solution (EDU:DMEM=1:1000) at 37°C for 2 hours. Rinse cell samples three with PBS. Fix cell sample with a freshly prepared 4% paraformaldehyde for 30 minutes at room temperature. Incubate cells with glycine solution for 5 minutes. Rinse cell samples three with PBS. Incubate cells with EDU reaction mixture at room temperature for 30 minutes. Rinse cell samples with 0.5% TritonX-100 three times. Incubate cells with F solution (F:ddH20=1:100) for 30 minutes. Rinse cell samples three with PBS. Samples can directly be analysed under a fluorescence microscope.

### Colony formation

Seed cells at 6-well plate each well 100cells or 500cells. Culture for three weeks. Fix cell sample with a freshly prepared 4% paraformaldehyde for 30 minutes. Rinse cell samples three with PBS. Stain with crystal violet for 10 minutes. Rinse cell samples three times with ddH_2_0.

### Statistic analysis

Specific to clinic samples, Kaplan–Meier curves for overall survival and TNM stage situation of the cohort of 96 patients stratified into the high DJ-1and low DJ-1 two groups based on DJ-1 IRS. Statistics of the curves were calculated using log-rank Mantel–Cox tests and the statistics of the TNM stage were calculated using Student t test. Other results were presented as mean ± SEM from at least 3 independent biological replicated experiments. Data were analyzed using Student t test.*P<0.05; **P<0.01; ***P<0.001.

## SUPPLEMENTARY MATERIALS FIGURES AND TABLES


