# Supplementary material for: DJ-1 promotes development of DEN-induced hepatocellular carcinoma and proliferation of liver cancer cells
Source: Oncotarget. 2016 Dec 27;8(5):8499–511. doi: 10.18632/oncotarget.14293 (PMC5352417; doi:10.18632/oncotarget.14293)
Supplement: Supplementary file 1 [file oncotarget-08-8499-s001.pdf]

DJ-1 promotes development of DEN-induced hepatocellular carcinoma and proliferation of liver cancer cells

SUPPLEMENTARY FIGURES AND TABLE

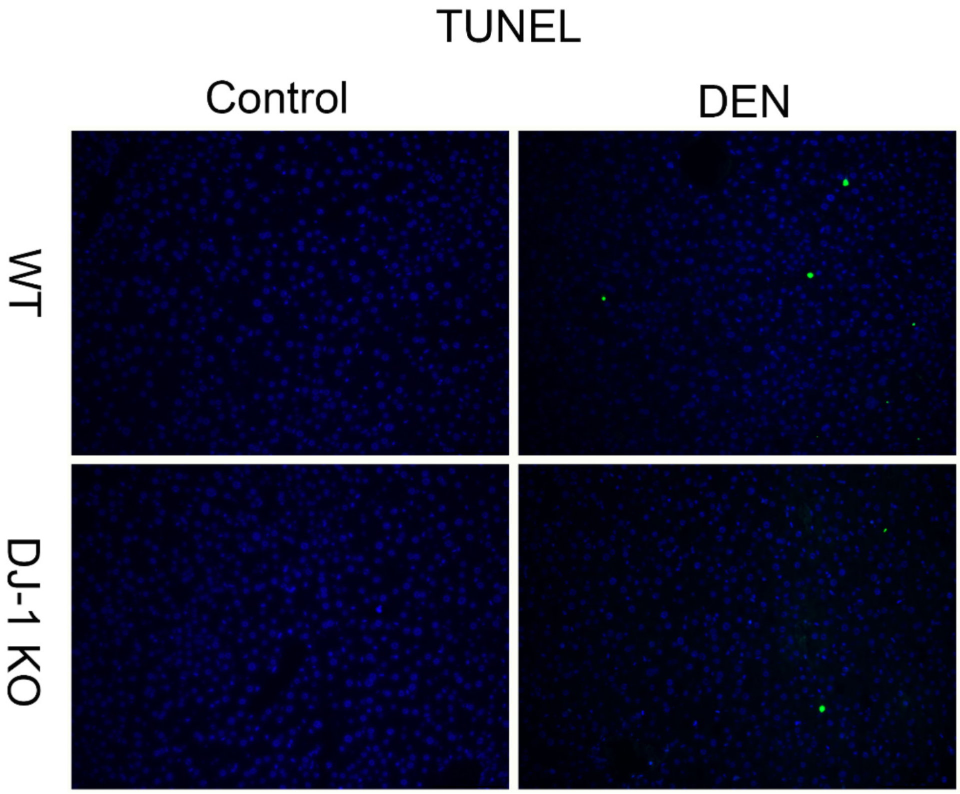

Supplementary Figure 1: DNA damage was analyzed by IHC TUNEL staining in HCC model.

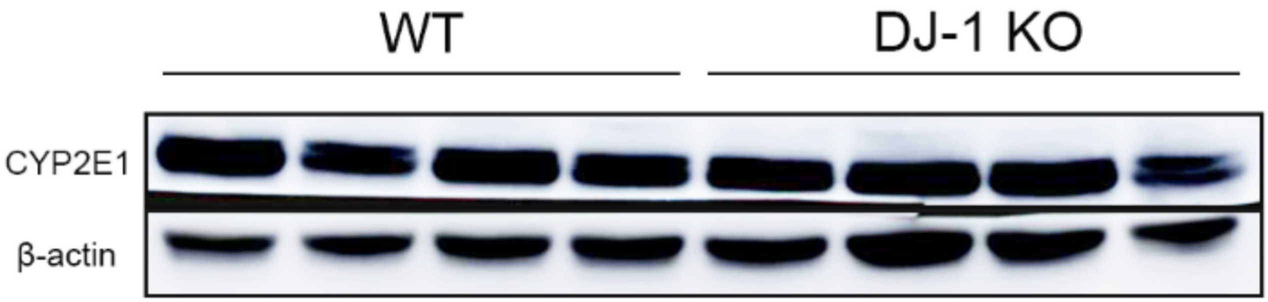

Supplementary Figure 2: cyp2E1 expression was examined in the liver from WT and KO in DEN acute model.

Supplementary Table 1: Demograhic,clinical characteristic, biochemical characteristic of 96 Patients

Characteristic	Low DJ-1	High DJ-1	p
No. of Patients	n=57	n=39	
Age			NS
mean±SD	54±11	52±11	
Gender			NS
Male	44(77.19%)	32(82.05%)	
Female	13(22.81%)	7(17.95%)	
Cirrhosis			NS
NO	15(26.32%)	5(12.82%)	
YES	42(73.68%)	34(87.18%)	
ALT(IU/L)			NS
Median	31	36	
Range	10-115	10-79	
AST(IU/L)			NS
Median	33	40	
Range	15-116	16-148	
TB(μmol/L)			NS
Median	17.8	16.2	
Range	4.6-65.9	6.8-46.6	
DB(μmol/L)			NS
Median	5.2	4.8	
Range	2.0-38.6	2.7-14.9	
AFP(ng/ml)			NS
Median	101.4	208.9	
Range	1.9-3000	1.4-3000	
CEA(ng/ml)			NS
Median	2.52	1.82	
Range	0.58-53.71	0.63-11.25	
HBeAg			NS
Negative	9(15.79%)	5(12.82%)	
Positive	48(84.21%)	34(87.18%)	

AFP, α-fetoprotein; ALT, Alanine Aminotransferase; AST, Aspartate Aminotransferase; HBeAg, hepatitis B e antigen; SD, standard deviation.
